# Steady-state estradiol triggers a unique innate immune response to allergen resulting in increased airway resistance

**DOI:** 10.1186/s13293-022-00483-7

**Published:** 2023-01-06

**Authors:** Kristi J. Warren, Cassandra Deering-Rice, Tom Huecksteadt, Shubhanshi Trivedi, Alessandro Venosa, Christopher Reilly, Karl Sanders, Frederic Clayton, Todd A. Wyatt, Jill A. Poole, Nicola M. Heller, Daniel Leung, Robert Paine

**Affiliations:** 1 George E Wahlen Salt Lake City VA Medical Center, 500 Foothill Dr., Salt Lake City, UT USA; 2 The Division of Respiratory, Critical Care, and Occupational Pulmonary Medicine, Department of Internal Medicine, University of Utah School of Medicine, Salt Lake City, UT USA; 3 Department of Pharmacology and Toxicology, College of Pharmacy, University of Utah, Salt Lake City, UT USA; 4 Division of Allergy and Immunology, Department of Internal Medicine, University of Nebraska Medical Center, Omaha, NE USA; 5 Department of Pathology, University of Utah, Salt Lake City, UT USA; 6 Veterans Affairs Nebraska-Western Iowa Health Care System, Omaha, NE USA; 7 Department of Anesthesiology and Critical Care Medicine, School of Medicine, Johns Hopkins University, Baltimore, USA; 8 The Division of Infectious Diseases, Department of Internal Medicine, University of Utah School of Medicine, Salt Lake City, UT USA

## Abstract

**Rationale:**

Asthma is a chronic airway condition that occurs more often in women than men during reproductive years. Population studies have collectively shown that long-term use of oral contraceptives decreased the onset of asthma in women of reproductive age. In the current study, we hypothesized that steady-state levels of estrogen would reduce airway inflammation and airway hyperresponsiveness to methacholine challenge.

**Methods:**

Ovariectomized BALB/c mice (Ovx) were implanted with subcutaneous hormone pellets (estrogen, OVX-E2) that deliver consistent levels of estrogen [68 ± 2 pg/mL], or placebo pellets (OVX-Placebo), followed by ovalbumin sensitization and challenge. In conjunction with methacholine challenge, immune phenotyping was performed to correlate inflammatory proteins and immune populations with better or worse pulmonary outcomes measured by invasive pulmonary mechanics techniques.

**Results:**

Histologic analysis showed an increase in total cell infiltration and mucus staining around the airways leading to an increased inflammatory score in ovarectomized (OVX) animals with steady-state estrogen pellets (OVX-E2-OVA) as compared to other groups including female-sham operated (F-INTACT-OVA) and OVX implanted with a placebo pellet (OVX-Pl-OVA). Airway resistance (Rrs) and lung elastance (Ers) were increased in OVX-E2-OVA in comparison to F-INTACT-OVA following aerosolized intratracheal methacholine challenges. Immune phenotyping revealed that steady-state estrogen reduced CD3+ T cells, CD19+ B cells, ILC2 and eosinophils in the BAL across all experiments. While these commonly described allergic cells were reduced in the BAL, or airways, we found no changes in neutrophils, CD3+ T cells or CD19+ B cells in the remaining lung tissue. Similarly, inflammatory cytokines (IL-5 and IL-13) were also decreased in OVX-E2-OVA-treated animals in comparison to Female-INTACT-OVA mice in the BAL, but in the lung tissue IL-5, IL-13 and IL-33 were comparable in OVX-E2-OVA and F-INTACT OVA mice. ILC2 were sorted from the lungs and stimulated with exogenous IL-33. These ILC2 had reduced cytokine and chemokine expression when they were isolated from OVX-E2-OVA animals, indicating that steady-state estrogen suppresses IL-33-mediated activation of ILC2.

**Conclusions:**

Therapeutically targeting estrogen receptors may have a limiting effect on eosinophils, ILC2 and potentially other immune populations that may improve asthma symptoms in those females that experience perimenstrual worsening of asthma, with the caveat, that long-term use of estrogens or hormone receptor modulators may be detrimental to the lung microenvironment over time.

## Highlights

- Steady levels of estrogen are associated with changes in allergic inflammation.
- When E2 is maintained at a level comparable to the mid-follicular phase of ovulation mucus production and airway hyperreactivity are increased.
- Estrogen reduces eosinophils and ILC2 following allergen challenges.
- Neutrophils and CD19+ B cells were unaffected in the lung tissues by estrogen treatment.

## Introduction

Asthma is a chronic respiratory disease that afflicts approximately 350 million people worldwide. Many of these individuals present in clinics and emergency rooms with a range of symptoms that include chronic coughing, frequent night awakenings, and excess mucus production [1, 2]. More severe disease is associated with airway constriction, shortness of breath and ultimately reduced FEV_1_ and FVC readings [1, 3]. While genetics, ethnicity, co-morbidities (e.g., diabetes and obesity) [4–6], and environmental exposures play a role in the development of asthma [7], biological female sex is correlated with severe asthma in adulthood [8–11].

Asthma is a complicated airway disease classically characterized as a type 2 immune response to allergen. In recent years it was established that ILC2, or group 2 innate lymphoid cells, play a central role in allergic responses [12]. ILC2 were initially characterized as cells capable of producing IL-5 at robust enough levels to recruit and activate eosinophils [13, 14]. Moreover, ILC2 contribute to M2 macrophage polarization in resident alveolar macrophages through IL-13 production, and contribute to chemotaxis of allergy-associated immune cells by producing chemokines (e.g., CCL17, CCL22) [12, 14–16]. IL-13 and IL-4 are produced by Th2 cells as well, but it is likely that those cells are programmed to some extent by ILC2 [17, 18]. Classical type 2 asthma responses lead to mucus production in the airways and IgE responses, however Th1 and Th17 CD4+ cells and corresponding immune responses have been characterized for subsets of asthmatics as well [19, 20]. Th1 cells may reduce airway hyperactivity and mucus production in animals [21, 22], usually characterized in viral-induced asthma exacerbation models, but the opposite is shown for IL-17. Th17 responses in animals sufficiently induce mucus in the airways [23], drive neutrophil recruitment and increase airway resistance [24]. Altogether there is still much left to uncover for this nebulous airway disorder, but it is likely that overlap between type 1, type 2 and type 17 immune phenotypes maybe driven by hormones or changes in circulating hormones in female asthmatics.

The overarching theme in sex-biased asthma is that ovarian hormones play a role in the development of severe asthma in women of reproductive age. During menstruation–ovulation cycling (day 0–14) estrogen serum concentrations vary between 20 and 500 pg/mL among women. Progesterone and estrogen serum concentrations increase simultaneously in females leading up to menstruation [P4; 7–15 ng/mL and E2; 100–300 pg/mL] (i.e., day 15–day 28), then drop rapidly to baseline levels [P4; 1.5 ng/mL E2; < 20 pg/mL] [25, 26]. While peak levels of the ovarian hormones are thought to exacerbate asthma symptoms in 30–40% of women around menstruation [26], it has also been postulated that the sudden drop in circulating progesterone and estrogen may explain increased asthma symptomology during that time [26]. In longitudinal studies, estrogen-based oral contraceptives are linked to reduced onset of asthma in women of reproductive age [27–29]. Along this line of reasoning estrogen-based oral contraceptives yield serum concentrations of estrogen equivalent to that of the mid-follicular phase of ovulation [5–80 pg/mL] [30–32]. This concentration is moderately low in comparison to the levels reached closer to ovulation or menstruation. Short-term and long-term hormone treatments need detailed investigation in asthmatic women as modulating hormones may be a safe strategy for regulating exacerbations in those subsets of asthmatic women who are sensitive to those hormone fluctuations.

Animal modeling is useful to understand hormone mechanisms that govern biological sex differences in male versus female asthmatics [8–11, 33–37]. Foundational studies show that aspects of inflammatory signaling pathways, microRNA expression, and precursor myeloid and lymphoid cell progenitors in the bone marrow are influenced by hormone receptor ligation that ultimately influences the lung response to allergen [19, 20, 34, 38]. In the present studies we examined steady-state estrogen (68.2 pg/mL ± 2.0) in the OVA-induced allergic inflammation model. To our surprise, estrogen had a profound effect on animals that underwent pulmonary function testing by making those animals more responsive to methacholine and thereby increasing airway hyper-reactivity readings. Based on these findings, we hypothesized that type 2 immunity (eosinophils and CD4+ Th2 cells) would increase with estrogen treatment, however, this was not the case. In fact, estrogen was associated with decreased type 2 cells and allergic inflammatory cytokines and chemokines in the BAL fluid (airways), but not lung tissue. Estrogen significantly increased MPO, the neutrophil chemokine (KC) and neutrophils in the lung tissue, indicating perhaps a shift towards a Th1 or Th17 phenotype in those animals treated steadily with estrogen. The study highlights distinct roles of estrogens on lung cells and immune cells in female-biased allergic responses.

## Materials and methods

### Mice, ovariectomies and subcutaneous hormone pellets

BALB/c mice were ovarectomized at 3 weeks of age at The Jackson Laboratory (Bar Harbor, ME). After 2 weeks of post-operation recovery, animals were transported to the animal facility where they acclimated for 1 week prior to beginning the experimental protocol. Deep anesthesia with achieved using ketamine [100 mg kg^−1^] and xylazine [16 mg kg^−1^] and implantation of a subcutaneous, 60-day, slow-release pellet containing estrogen (E2-17β; 0.1 mg) or placebo pellets purchased from Innovative Research of America (Sarasota, FL, USA). Female, sham-operated animals were included as controls (F-INTACT). Post-surgery animals were given two doses of buprenorphine 12 h apart for pain management according to standard protocols. Two weeks after surgery, animals were sensitized with ovalbumin (Grade V, 500 µg/mL) and aluminum hydroxide (Sigma, St. Louis, MO; 20 µg/mL) at 3-week intervals followed by 5 daily challenges with intranasal 1.5% ovalbumin in sterile saline; according to a previously used protocol [39, 40]. All protocols were approved by the IACUC or Research Advisory Committees at the University of Nebraska Medical Center (Omaha, NE) or the Salt Lake City VA Medical Center (Salt Lake City, UT).

### Bronchoalveolar lavage fluid collection and analysis

Euthanized animals were placed in the prone position and small incision was made to expose the trachea, followed by a 1–3 mM width incision made to the trachea for cannulation. A 1-mL syringe was filled and fitted onto the cannula and the lungs were slowly flushed three times with 1 mL of Dulbecco’s PBS. The first wash was collected into a separate tube and centrifuged at 300×*g* for 10 min at 4 °C. After centrifugation the supernatants were separated from cell pellets for cytokine and chemokine analysis by ELISA. The second and third washes were collected and centrifuged 300×*g* to separate cellular content from the supernatants; the cellular pellets from the three washes were combined and total cells were counted using the TC-20 automated counter (Biorad) with trypan blue exclusion. Approximately 20–40,000 cells were applied to cytospin slides and stained with Giemsa for cell differential determination; Giemsa staining procedure, previously described [41].

### Lung histology and scoring

Chest cavity was exposed following deep anesthesia and lungs were cleared of blood by cardiac perfusion with saline solution. Whole lungs were fixed by tracheal instillation of 10% neutral buffered formalin at a constant pressure (25 cmH_2_O). Following paraffin embedding, 6 µm sections were cut and stained with hematoxylin and eosin (H&E) and periodic-acid Schiff staining (PAS) by the Associated Regional and University Pathologists Inc., at the University of Utah. Image acquisition occurred the following day to allow the mounting reagent to fully dry. Images (100× and 200× magnification) were acquired using a Zeiss Axioscope 7 (Carl Zeiss Meditec, Inc., Dublin, CA) and processed using Adobe Photoshop (San Jose, CA) [41]. Inflammation scores were calculated as follows: (% of bronchial/bronchiolar epithelium with infiltrate × measured number of cellular depth of peribronchial infiltrate) + (% of pulmonary veins with infiltrate × measured number of cellular depth of perivenous infiltrate). This score was calculated on 2 slides per animal and 3–5 animals per group. Representative images are shown in Fig. 2 at 100× and 200× magnification. A scoring method was developed previously to quantify the degree of PAS + staining in the moderate to larger airways [15]. Briefly, the percentage of each airway is assigned an arbitrary score: 0 = 0–25% PAS+, 1 = 25–50% PAS+, 2 = 50–75% and 3 = 75–100%. The mean cumulative score for each group is determined and evaluated by statistical method for significance.

### ELISA

Cell culture supernatants BAL fluids, and serum were centrifuged at 400×*g* at room temperature for 10 min to clear cellular debris prior to testing. IL-5, IL-13, CCL3, CCL11, CCL17, CCL22 and KC Duo-set ELISA (R&D Systems, Minneapolis, MN) were performed according to the manufacturer’s instructions. Absorbance was measured on the SpectraMax M3 (Molecular Devices LLC, San Jose, CA) at 450 nm with 570 nm wavelength correction. ILC2 culture supernatants were diluted at least 1:10 with reagent diluent provided with the Ancillary Reagents Kit II (R&D Systems) that accompanies the ELISA Duo-set kits. Estrogen was measured by ELISA according to the manufacturer’s instruction after a 50-fold dilution in reagent buffer provided in each kit (Abnova, Taipei, Taiwan). OVA-specific IgE was measured in serum according to the manufacturer’s instructions (Cayman Chemical, Ann Arbor, Michigan).

### Pulmonary mechanics

BALB/c mice (*n* = 4–7/group) were used to assess pulmonary function using a FlexiVent FX-1 small animal ventilator (Flexivent FX1; SCIREQ Inc., Montreal, Qc, Canada). We measured airway resistance (Rrs), compliance (Crs), and elastance (Ers) in anesthetized (ketamine + xylazine 100 + 20 mg/kg), paralyzed (0.1 mg/kg vecuronium bromide), and tracheostomized mice as described previously [42, 43]. Baseline measurements were collected using broadband low-frequency forced oscillations for each mouse. This was followed by assessment of bronchial reactivity/contractility elicited by delivery of aerosolized methacholine at 12.5 mg/mL using the Flexivent Aeroneb fine particle nebulizer in saline for 10 s per dose at 4–5 min intervals. The maximum response for measured criteria was determined at baseline, after saline and after each methacholine nebulization. Data are represented as methacholine responses (12.5 mg/mL) after baseline subtraction. Baseline PV-loops were generated in seven equal steps between 3 and 30 cmH_2_O and normalized to the individual mouse weight.

### ILC2 isolation and cell culture conditions

Lungs were perfused with 2 mL of 1× Dulbecco’s PBS, excised and digested in Hank’s Balanced Salt Solution containing Collagenase, Type I [10 µg/mL] (Worthington Biochemical, Lakewood, NJ) and DNase I [0.02 mg/mL] (Sigma-Aldrich) for 30 min at 37 °C. Single-cell suspensions were passed through 40-micron nylon filters and washed one time with PBS containing 2% FBS. ILC2 were enriched from single cell suspensions using the mouse ILC2 enrichment kit (StemCell Technologies, Vancouver, Canada) followed by labeling with anti-mouse antibodies against CD45, CD3, B220, CD11c, CD11b, F4/80, Ter-119, Ly6c, Ly6g, CD127, CD25, KLRG-1 and ICOS. ILC2 purification was completed by FACS sorting on the ARIA II (BD Biosciences, Franklin Lakes, NJ). We acquired approximately 35,000–50,000 purified ILC2 from 4 to 5 ovalbumin-treated control animals, or approximately 10,000–15,000 purified ILC2 from 6 to 8 naïve male and female mice. Purified ILC2 were split equally across 4–6 wells of round-bottom 96-well plate (Corning, Corning, NY), followed by the addition of IL-2 and IL-7 with and without IL-33 for 72 h in culture. Viability was assessed by trypan blue exclusion on the TC-20 cell counter (Biorad, Hercules, CA). Cell culture supernatants were collected and stored at − 80 °C until analysis by ELISA could be completed.

### Statistical analysis

One-way ANOVA was used to determine statistical differences among groups; between groups comparisons were made using the Tukey’s post-test. A *p*-value less than 0.05 was considered significant. All statistical analysis was performed using the statistics software incorporated into GraphPad Prism, Version 10 (La Jolla, CA).

## Results

### Airway and vascular cell infiltrates are increased in the airways when estrogen is given at steady-state levels

**Fig. 1:**
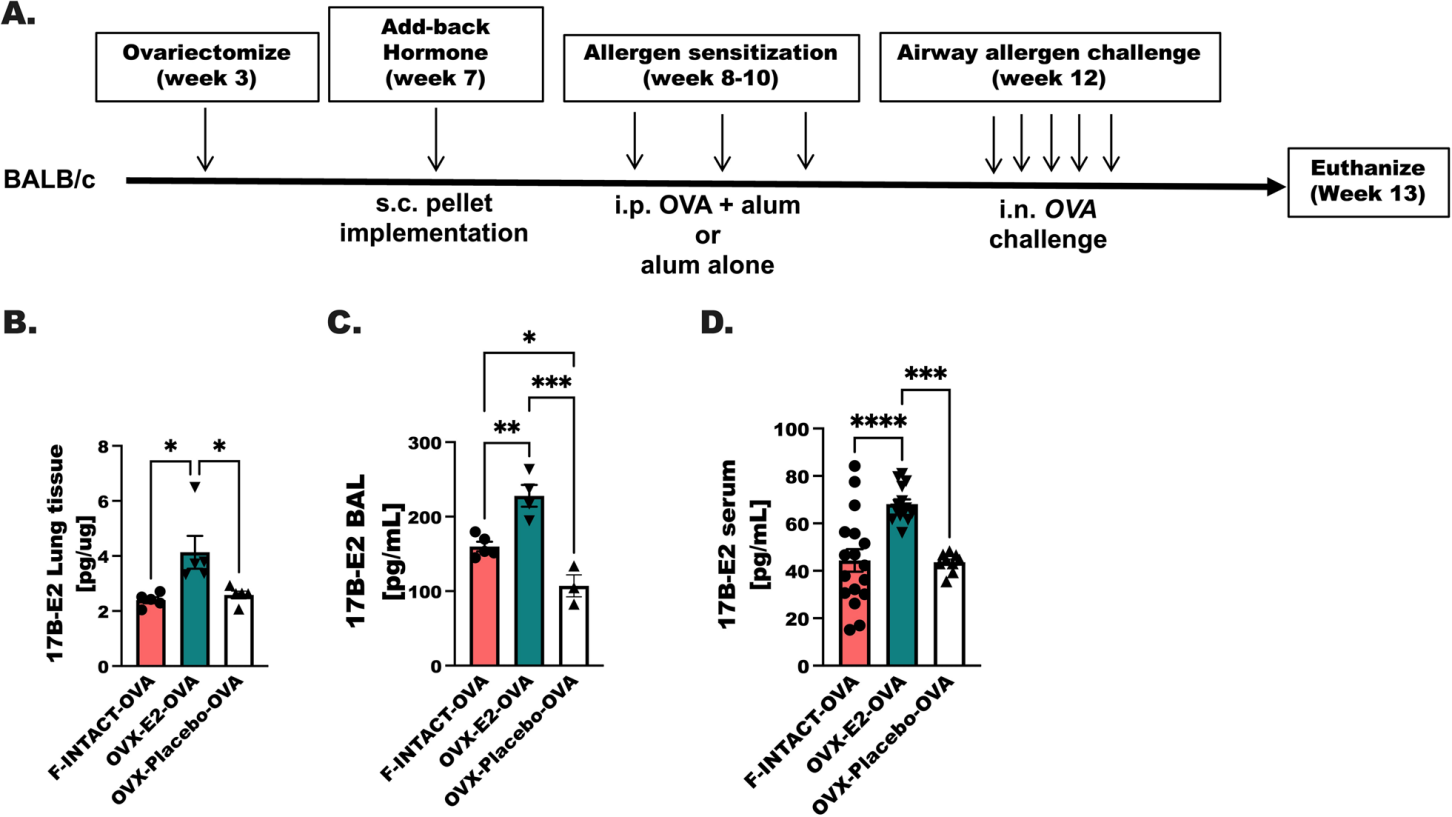
Airway and vascular cell infiltrates are increased in the airways when estrogen is given at steady-state levels. **A** An overview our experimental protocol is shown where female BALB/c mice were ovarectomized at 3 weeks of age (OVX) and implanted with subcutaneous estrogen (17β-E2, 0.1 mg) or placebo pellets. Sham-operated females (F-INTACT) were included as controls. OVA-treated animals were sensitized to chicken egg albumin by intraperitoneal injections with OVA and aluminum hydroxide (100 µL). Saline treated animals were included as controls. **B**–**D** Levels of estrogen were determined by ELISA in lung tissue, BAL and serum. One-way ANOVA were used to determine statistical differences followed by Tukey’s post-test to determine between groups differences. Statistical significance was assigned when *p*-value was less than 0.05; *p < 0.05, **p < 0.01; ***p < 0.001; ****p < 0.0001

Adult females are more likely to have increased allergic inflammation in comparison to men, yet steady-state levels of estrogen in the form of oral contraceptives improve asthma severity in adult women [27, 28, 44]. With these data in mind, we hypothesized that estrogen may improve allergic inflammation in allergen-challenged animals. Ovarectomized animals (OVX) were implanted with subcutaneous estrogen (OVX-E2) or placebo pellets (OVX-Placebo) and allergic inflammation was experimentally generated using chicken egg ovalbumin (OVA) sensitization and airway challenge protocols (Fig. 1A). Higher levels of estrogen were appropriately detected in the lung tissue, BAL and serum of ovarectomized animals implanted with an estrogen pellet, while reduced levels of estrogen were detected in Female-INTACT animals treated with OVA or in OVX animals that were implanted with a placebo pellet (Fig. 1B–D).

**Fig. 2:**
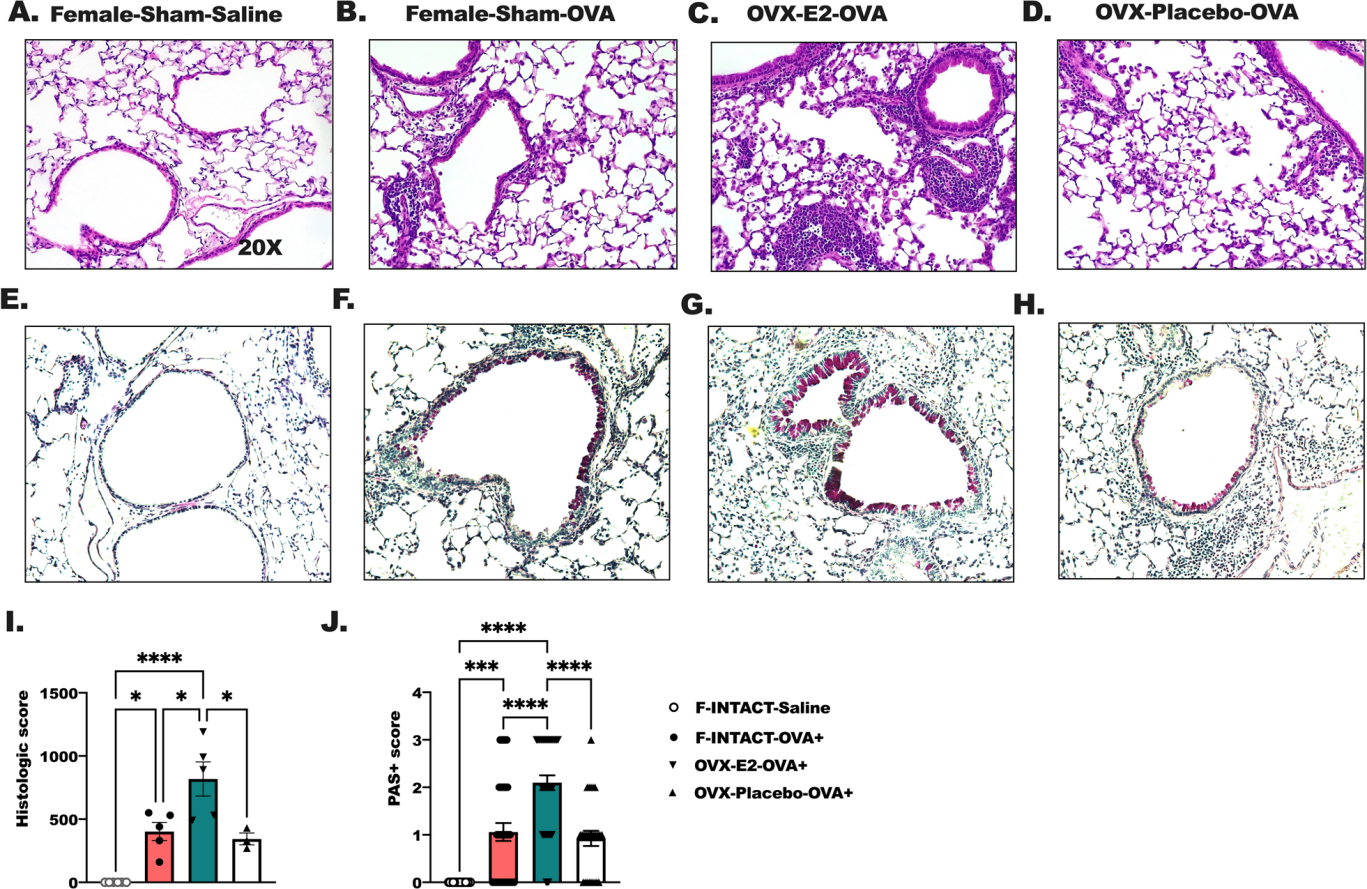
Airway resistance is increased in ovarectomized animals treated with estradiol in comparison to female INTACT, OVA-treated animals. After five daily airway challenges with i.n. ovalbumin, lungs were inflated then excised followed by paraffin embedding and H&E staining. **A**–**I** Histological scoring was performed by a blinded pathologist on two sections per animal, 3–4 animals per group. The data are shown as mean ± SEM. Results represent 2 independent experiments. One-way ANOVA were used to determine statistical differences followed by Tukey’s post-test to determine between groups statistical effects. Statistical significance was assigned when *p*-value was less than 0.05; *p < 0.05, **p < 0.01; ***p < 0.001; ****p < 0.0001

The level of cellular immune infiltration was determined by quantifying bronchial and vascular inflammation and cellular infiltrates (Fig. 2A–D, I). No inflammation was found in the F-INTACT-saline-treated animals even after methacholine challenge (Fig. 2A). F-INTACT-OVA had more inflammation than Female-INTACT-Saline mice indicating a significant induction (*p* < 0.05) of allergic inflammation with OVA sensitization and airway challenges (Fig. 2A, B). OVX-E2-OVA also had more inflammatory cell infiltrate than F-INTACT-OVA-treated when comparing inflammatory scoring results (*p* < 0.05) (Fig. 2B, C, I). Mucus production was assessed using PAS staining (Fig. 2E–H, J). PAS staining was statistically higher in F-INTACT-OVA mice compared to saline-treated animals (*p* < 0.001), and OVX-E2-treated animals produced more mucus in airway cells in response to OVA than F-INTACT-OVA (*p* < 0.0001). In summary, these studies showed that estrogen significantly increased the allergic inflammatory response to airway allergen challenge.

### Airway resistance is increased in ovarectomized animals treated with estradiol in comparison to female INTACT, OVA-treated animals

**Fig. 3:**
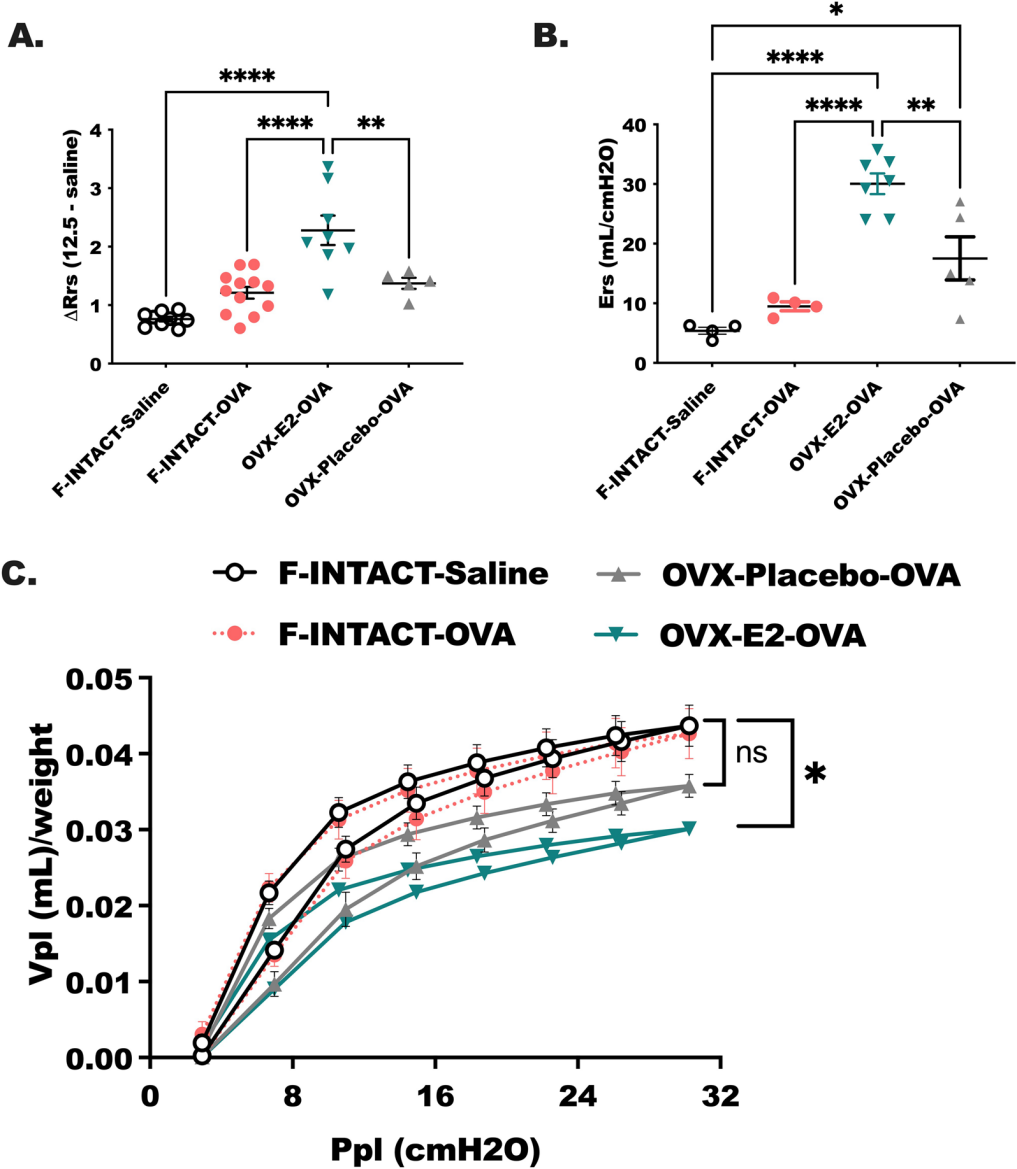
Airway resistance is increased in ovarectomized animals treated with estradiol in comparison to female INTACT, OVA-treated animals. After completion of the experimental protocol outline in Fig. 1 animals were subjected to methacholine challenge (12.5 mg/mL) using the flexivent system (Scireq). **A** Resistance (Rrs, baseline subtracted) and **B** elastance (Ers; baseline subtracted) were measured following methacholine challenge. **C** Pressure–volume loops were determined and normalized to body weight. The data are shown as means ± SEM. Results represent 3 independent experiments, *N* = 4 = 7/group. Mixed-ANOVAs were used to determine statistical differences using multiple variate selection followed by Sidak’s post-test (**C**), while a one-way ANOVA was used to determined statistical differences; as before a Tukey’s post-test was used to determine between group differences. Statistical significance was assigned when *p*-value was less than 0.05; *p < 0.05, ****p < 0.0001

In the next studies respiratory system mechanics were measured by determining resistance and elastance following methacholine challenge in each of the treatment groups: Female-INTACT-Saline, Female-INTACT-OVA, OVX-E2-OVA and OVX-Placebo-OVA (as in Fig. 1A). Airway resistance (Rrs) is an indicator of airway flow and airway obstruction, while elastance (Ers) determines the stiffness of the lung tissue. Rrs was higher in OVX-E2-OVA compared to Female-INTACT-OVA (Fig. 3A; *p* < 0.0001). Differences in Rrs between F-INTACT-Saline compared to F-INTACT-OVA did not reach statistical significance (*p* = 0.0876), but Rrs was higher in OVA-E2-OVA compared to OVX-placebo-OVA (*p* < 0.01). Elastance (Ers) readings trended upwards in F-INTACT-OVA animals compared to F-INTACT-Saline (*p* = 0.0845), but reached statistical significance in OVX-E2-OVA mice compared to F-INTACT-OVA mice (*p* < 0.001), and in OVX-E2-OVA compared to OVX-Placebo-OVA (Fig. 3B; *p* < 0.01). Pressure volume (PV) loops were also determined over the course of pulmonary function testing (Fig. 3C). We observed changes, or flattening, of the pressure–volume loops F-INTACT-OVA compared to OVX-E2-OVA (*p* < 0.05), which coincides with the increased Ers in OVX-E2-OVA mice compared to F-INTACT-OVA. No statistical differences were observed between OVX-placebo-OVA mice and F-INTACT-OVA. Altogether, the pulmonary function testing indicates that estrogen treatment stiffened the lung tissues in combination with increasing airway resistance in ovarectomized mice treated with ovalbumin.

### Total immune cell infiltrate is reduced in BAL fluid from OVX-E2-OVA-treated animals compared to F-INTACT-OVA-treated

**Fig. 4:**
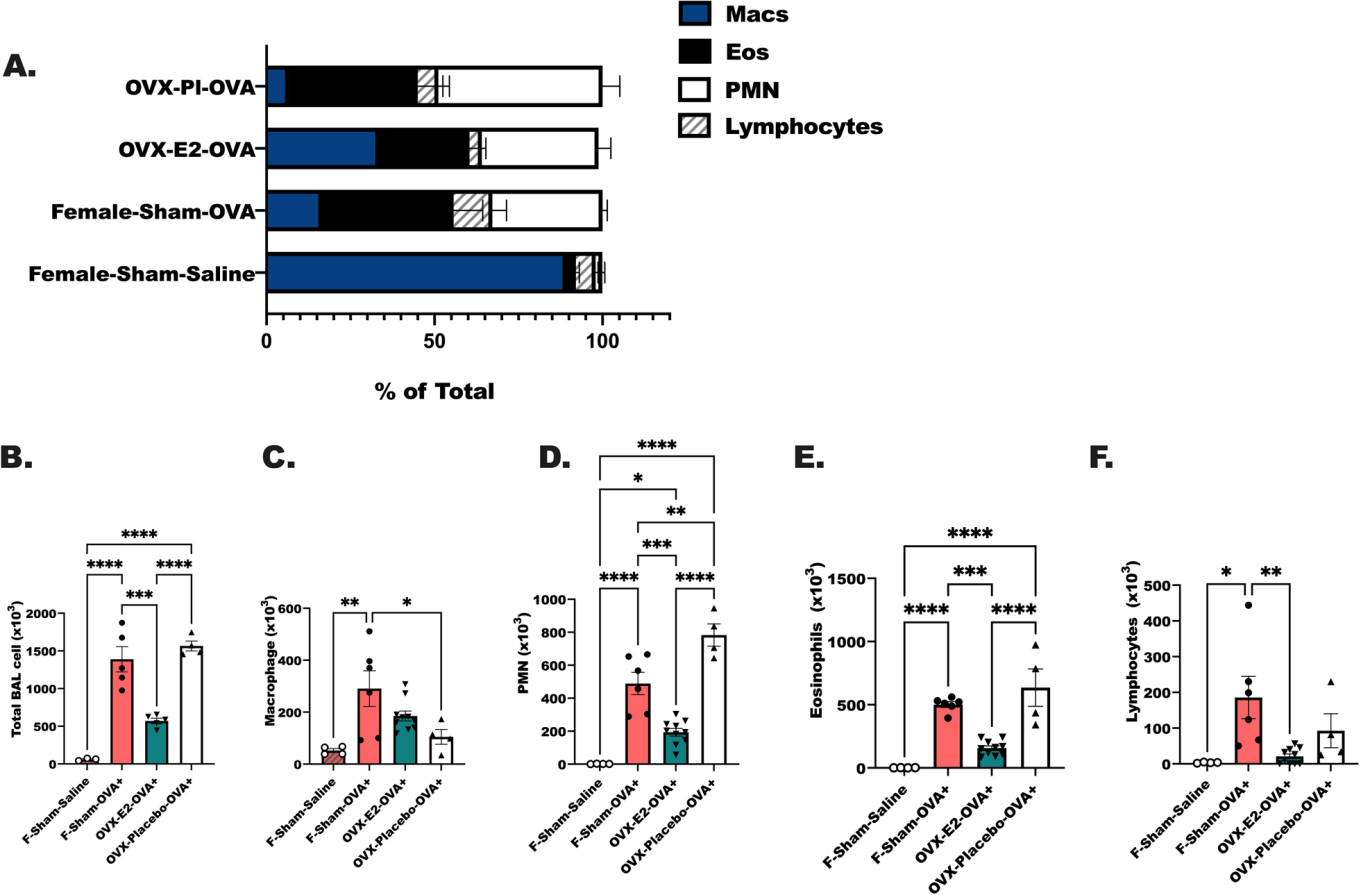
Total immune cell infiltrate is reduced in BAL fluid from OVX-E2-OVA-treated animals compared to F-INTACT, OVA-treated. Animals were treated as in Fig. 1. Cytospins were prepared from BAL fluids collected immediately following methacholine challenges. Total BAL return volume and numbers of total cells (**B**) were recorded and applied to cytospin slides at 300×*g* for 10 min. Slides were stained with Giemsa and 2–4 fields were assessed per slide. 200 cells per field were counted as **C** macrophages, **D** neutrophils, **E** eosinophils or **F** lymphocytes. **A** Percentages of each cell population are shown. **B**–**F** Are the counts of cells determined by multiplying the percentages by the total number of cells recovered in each lavage. Data are representative of 3 independent experiments with 3–8 animals per group. One-way ANOVA was used to determine statistical differences across all groups; Tukey’s post-test were used to determine statistical differences between groups. *p < 0.05, **p < 0.01, ***p < 0.001, ****p < 0.0001

We compared the composition of immune airway infiltrates in each treatment group following OVA treatments and methacholine challenge (as in Fig. 1A). All OVA-treated mice had significant increases in neutrophils and eosinophils in comparison to F-INTACT-Saline mice (Fig. 4A). OVX-E2-OVA-treated animals had a higher percentage of macrophages represented in the BAL cells collected as compared to F-INTACT-OVA, while OVX-placebo-OVA had a lower percentage of macrophages in comparison to F-INTACT-OVA. In addition, OVX-E2-OVA mice had a lower percentage of total lymphocytes as compared to F-INTACT-OVA. Counts for each population were determined using the volume of BAL recovered multiplied by the percentage of cell type counted per field (Fig. 4B–F). First, total BAL cells were reduced in OVX-E2-OVA-treated animals compared to F-INTACT-OVA (*p* < 0.001); OVX-placebo-OVA-treated mice maintained a comparable number of total cells to F-INTACT-OVA in their BAL (Fig. 4B). The counts of macrophages were reduced in OVX-Placebo-OVA compared to F-INTACT-OVA (*p* < 0.05), but macrophages were comparable in OVX-E2-OVA and F-INTACT-OVA (Fig. 3C). Most interestingly, OVX-E2-OVA had lower counts of neutrophils and eosinophils in comparison to F-INTACT-OVA (*p* < 0.001) (Fig. 4D, E), while OVX-Placebo-OVA mice had increased numbers of neutrophils in comparison to F-INTACT-OVA mice (*p* < 0.001). Finally, less lymphocytes were recovered from the BAL fluid of OVX-E2-OVA-treated animals compared to F-INTACT-OVA controls (*p* < 0.01) (Fig. 4F). Together these data suggest that estrogen reduced the airway-recruited immune cells that contribute to allergic outcomes.

### Allergic inflammatory cytokines in OVX-E2-OVA-treated animals in BAL and lung tissue compared to female intact mice treated with airway allergen

**Fig. 5:**
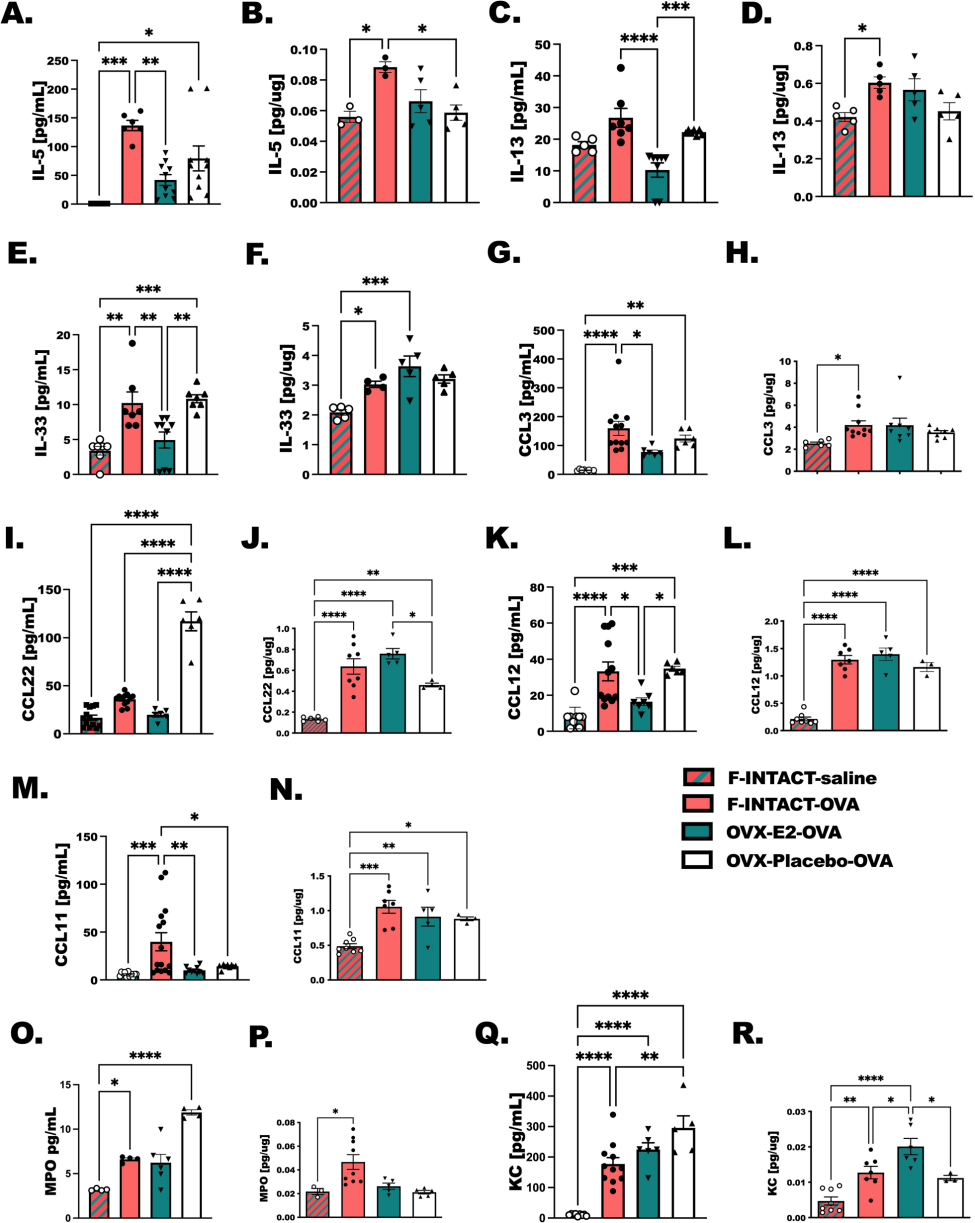
Allergic inflammatory cytokines in OVX-E2-OVA-treated animals in BAL and lung tissue compared to female intact mice treated with airway allergen. Animals were prepared as in Fig. 1. Animals were euthanized by overdosing with ketamine (200 µg/kg). Bronchoalveolar lavage fluid was collected and cleared by centrifugation (400×*g*) prior to applying to ELISA plates for analysis. **A**, **B** IL-5, **C**, **D** IL-13, **E**, **F** IL-33, **G**, **H** CCL3, **I**, **J** CCL22, **K**, **L** CCL12, **M**, **N** CCL11, **O**, **P** MPO, and **Q**, **R** KC are shown as pg/mL. Statistical significance was determined as in previous studies using a one-way ANOVA followed by Tukey’s post-test which are reported as *p* < 0.05 and considered significant in all studies. *p < 0.05, **p < 0.01, ***p < 0.001, ****p < 0.0001

To better understand the increased airway resistance in OVX-OVA-E2 mice, and reduced allergic cell populations in the BAL, we next compared BAL fluid cytokines and chemokines, to cytokines and chemokines detected in total lung tissue. IL-5, IL-13, IL-33, CCL3, CCL12, and CCL11 were decreased in OVX-E2-OVA animals compared to Female-INTACT-OVA in the BAL (Fig. 5A, C, E, G, and K), however we detected no differences between these two groups in the remaining lung tissues for these cytokines and CCL22 (Fig. 5B, D, F, H, J, L, N). MPO and KC were comparable in BAL from F-INTACT-OVA and OVX-E2-OVA (Fig. 5O, P), but KC was higher in OVX-E2-OVA compared to F-INTACT-OVA (*p* < 0.05) (Fig. 5R).

### Circulating cytokines and chemokines are selectively altered with steady-state estrogen treatment

**Fig. 6:**
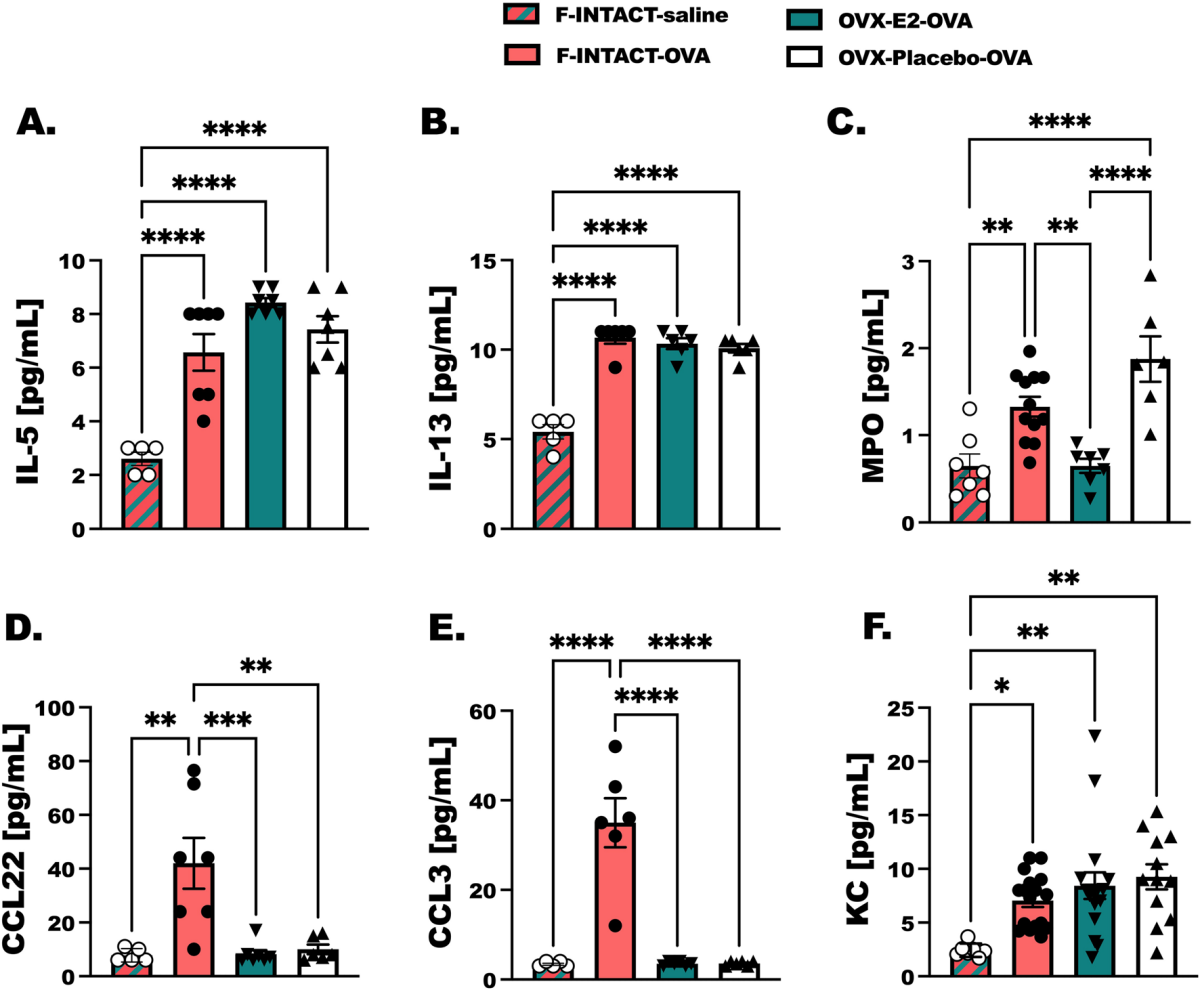
Circulating cytokines and chemokines are selectively altered with steady-state estrogen treatment. Groups of mice were prepared as in Fig. 1. Circulating cytokines were determined by ELISA in serum that was diluted 1:5 for all groups. **A** IL-5 and **B** IL-13 cytokines were detected, **C** the neutrophil activation marker, MPO, and **D**–**F** circulating chemokines **D** CCL22, **E** CCL3 and **F** KC are shown. The data are shown as mean ± SEM. Data are representative of 4 independent experiments. Statistical significance was determined as before with a *p* < 0.05 (indicated by *), **p < 0.01, ***p < 0.001, ****p < 0.0001

Serum was collected from OVA-treated animals for detection of circulating allergic-inflammatory cytokines and chemokines. Serum concentration of IL-5, IL-13, MPO, CCL22, CCL3, and KC were increased in F-INTACT-OVA mice compared to F-INTACT-saline mice (*p* < 0.05) (Fig. 6A–F). Similar to the findings in the BAL fluid, serum concentrations of CCL22 and CCL3 were decreased in OVX-E2-OVA mice as compared with F-INTACT-OVA mice (Fig. 6D, *p* < 0.001, Fig. 6E, *p* < 0.0001). MPO was also significantly reduced in serum from OVX-E2-OVA-treated animals compared to F-INTACT-OVA animals (Fig. 6C; *p* < 0.01). Although we saw reductions in neutrophil numbers in OVX-E2-OVA mice compared to F-INTACT-OVA in the previous studies (Fig. 4D), the neutrophil chemoattractant protein, KC, was not different between any of the OVA-treated groups.

### Estrogen treatment reduces eosinophils and neutrophils in the BAL of OVX-OVA-challenged mice

**Fig. 7:**
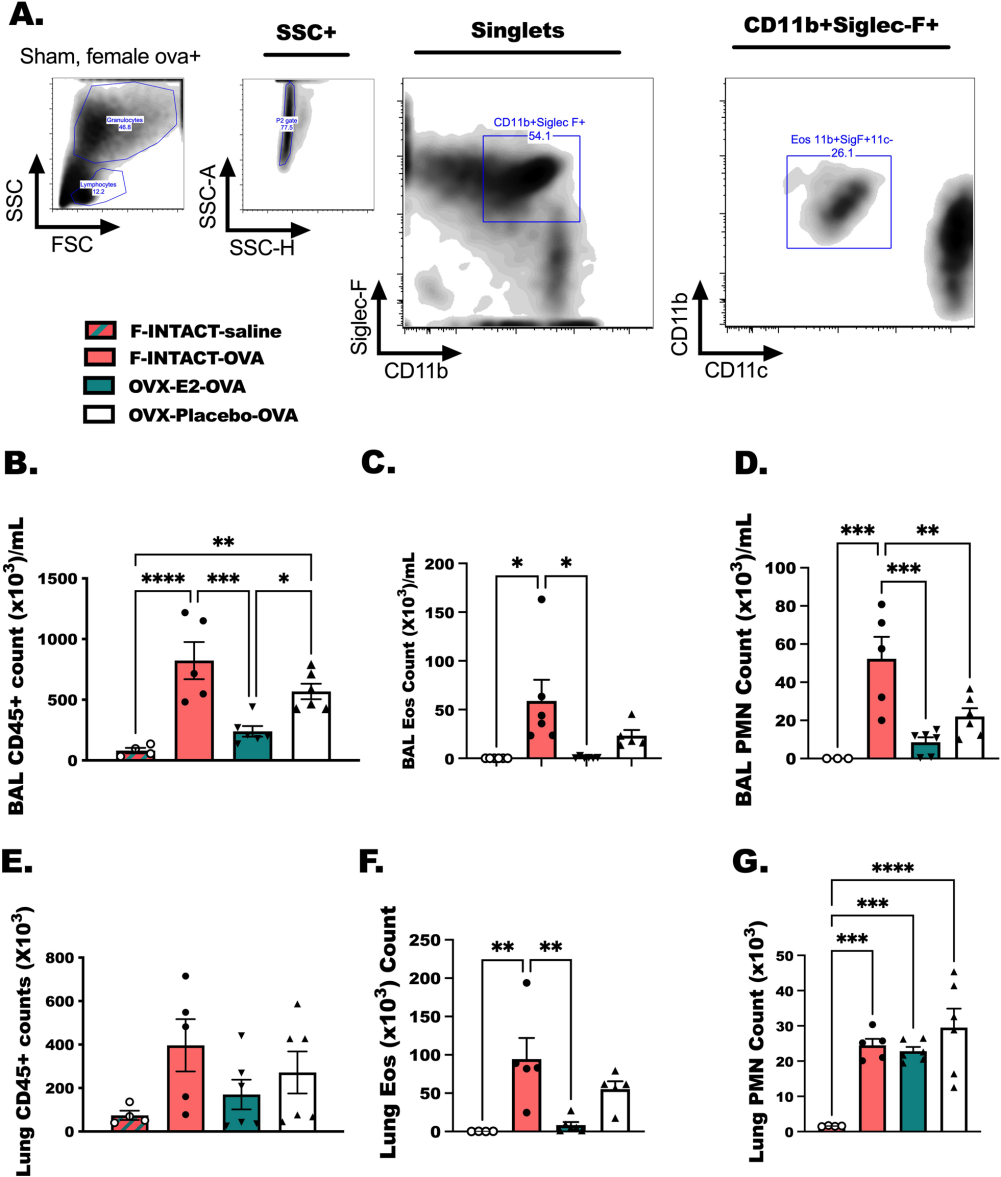
Estrogen treatment reduces eosinophils and neutrophils in the BAL of OVX-OVA-challenged mice. Animals were prepared as in Fig. 1. **A** Flow cytometry gating strategy for eosinophils defined as live, singlet, CD11b^+^Siglec-F^+^CD11c^−^ cells is shown, while neutrophils are defined as FSC^lo^SSC^hi^CD11b+CD11c+Siglec-F^lo^. **B** Total CD45+ BAL cells, **C** eosinophil and **D** neutrophils per mL of lavage fluid are shown for all groups. **E** Total CD45+ cells detected in the remaining lung tissue **F** eosinophils and **G** neutrophils were quantified. Bar graphs show the data as mean ± SEM (*n* = 5–6 mice/group). The data represent 4 independent experiments. Statistical significance was determined as in Fig. 3. * Indicates *p* < 0.05; **p < 0.01, ***p < 0.001, ****p < 0.0001

Eosinophils are regularly detected in circulation and in sputum of asthmatic patients [45, 46], as such eosinophils are thought of clinically as a determinant in asthma diagnosis and severity of disease. Other studies show that neutrophils are important mediators of airway and lung inflammatory responses [47, 48], and are capable of generating significant airway hyper-reactivity in methacholine-challenged animals [49]. In these studies, we prepared mice as in Fig. 1A and characterized eosinophil and neutrophil levels by flow cytometry in both BAL and remaining lung tissue (Fig. 7). As expected, increased numbers of CD45+ immune cells (*p* < 0.0001) (Fig. 7B, E) were detected in the BAL of OVA-treated animals compared to saline treated. Eosinophils (*p* < 0.05) (Fig. 7C, F) and neutrophils (PMN, *p* < 0.05) (Fig. 7D, G) were increased following OVA-allergen challenge in bronchoalveolar lavage fluids and in the lung tissue in comparison to saline-treated animals (*p* < 0.05). As with the previous data (Fig. 4), we again detected reduced numbers of eosinophils in OVX-E2-OVA-treated animals in comparison to F-INTACT-OVA (*p* < 0.05). Together this indicates a suppressive effect on eosinophils, again, a well-excepted biomarker of asthma, with pharmacologically delivered estrogen in the traditional OVA model. Finally, we detected no changes in total lung CD45+ cells or in lung tissue PMN when comparing F-INTACT-OVA to OVX-E2-OVA (Fig. 7E, G).

### Steady-state estrogen reduces airway and lung T cells, but CD19+ B cells are preserved and comparable to hormonally intact, female controls

**Fig. 8:**
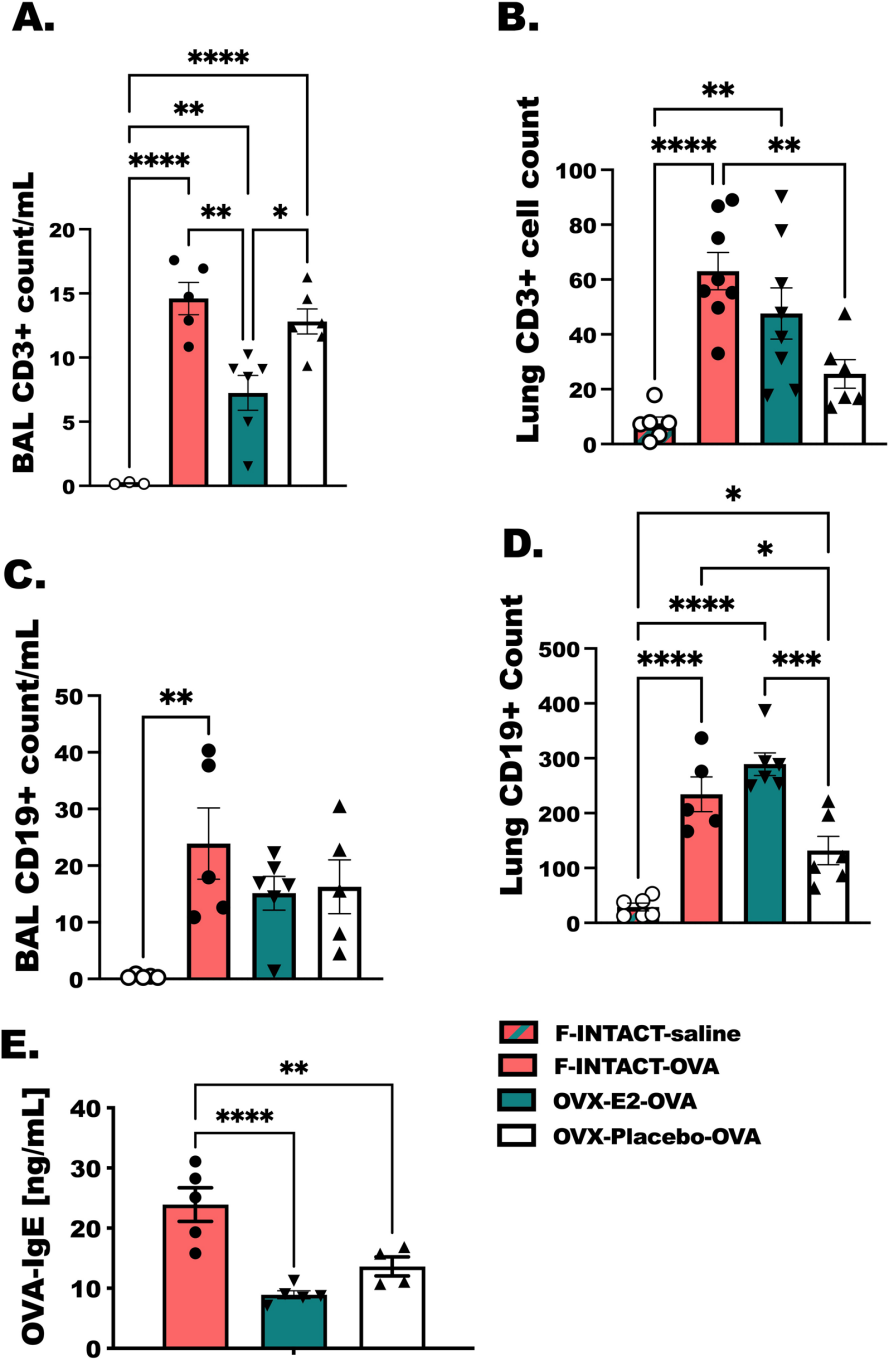
Steady-state estrogen reduces airway and lung T cells, but CD19+ B cells are preserved and comparable to hormonally intact, female controls. Groups of animals were prepared as in previous figures. Bronchoalveolar lavage fluid was collected immediately following euthanasia. **A**–**D** By flow cytometry total CD3+ and CD19+, T and B cells, respectively, were determined following treatment with 5 consecutive daily i.n. OVA administration. **A**, **C** Counts of T cells and B cells per mL of returned lavage fluid and **B**, **D** counts of T and B cells acquired from remaining lung tissue are shown. **E** OVA-specific IgE was also measure in serum that was diluted 1:5 by ELISA. Results are representative of two separate experiments with 4–6 animals included per group. As before, statistical significance was determined using one-way ANOVA with Tukey’s post-tests. As in previous figures * indicates *p* < 0.05; **p < 0.01, ***p < 0.001, ****p < 0.0001

Total BAL and lung CD3+ T cells (Fig. 8A, B) and CD19+ B cells (Fig. 8C, D) were assessed in the same treatment groups as before by flow cytometry. F-INTACT-OVA had higher numbers of CD3+ T cells detected in the BAL and lung in comparison to Female-INTACT-Saline (*p* < 0.0001), however total CD3+ T cells were significantly reduced in OVX-E2-OVA mice compared to F-INTACT-OVA mice in BAL only. CD19+ B cells (Fig. 8C, D) were not significantly reduced in the BAL or lung tissue from OVX-E2-OVA animals as compared with F-INTACT-OVA mice. IgE levels are determinant of degree of allergic responses in the clinic and typically determined as another prototypical biomarker of asthmatic disease. Although B cells responses did not reach statistical reductions in OVX-E2-OVA compared to F-INTACT-OVA, we did find less OVA-specific IgE with estrogen treatment (*p* < 0.0001) (Fig. 8E).

### Accumulation and IL-33-induced activation of lung ILC2 is reduced in OVX-E2-OVA mice compared to F-INTACT-OVA mice

**Fig. 9:**
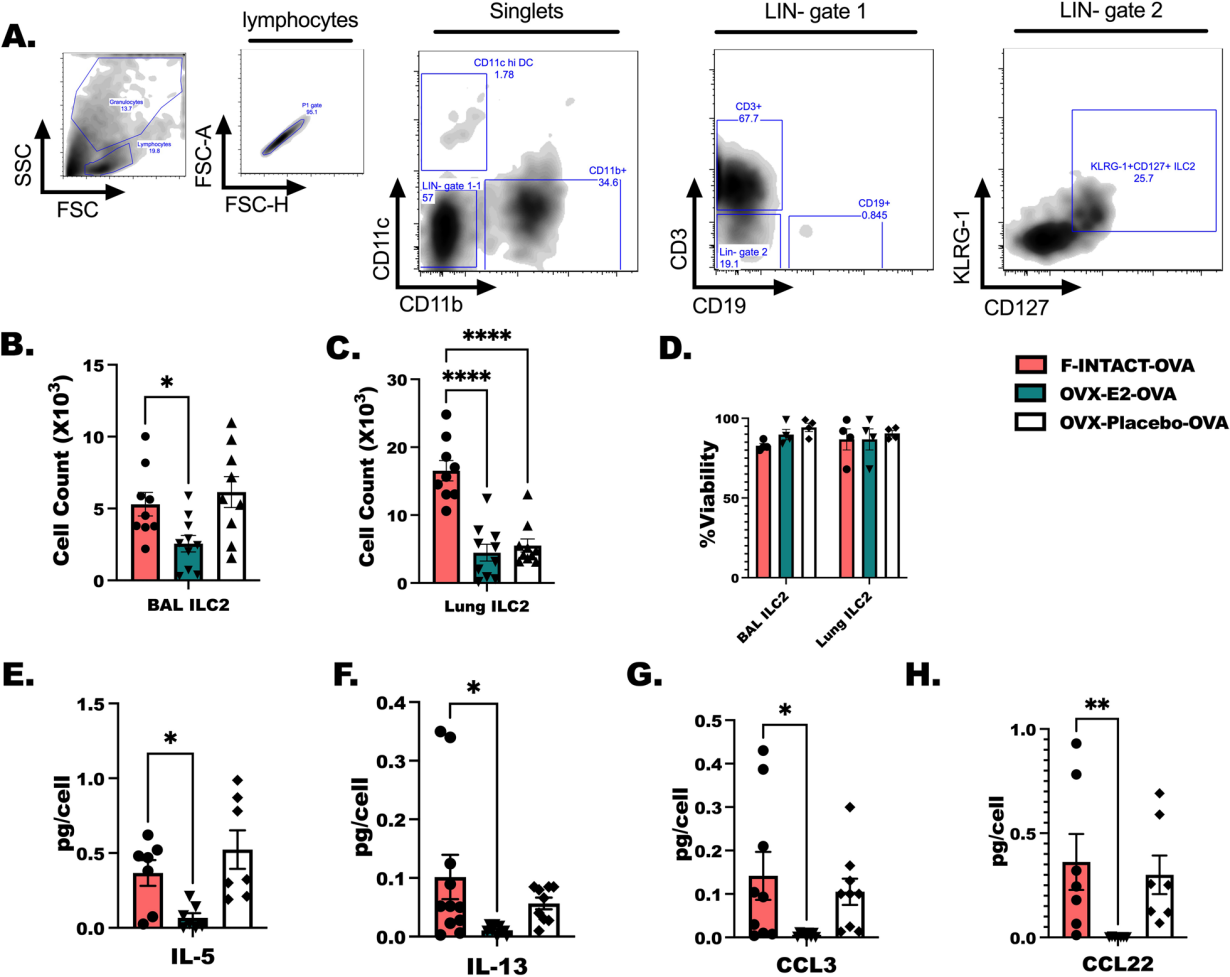
In vivo estrogen treatment reduces ILC2 numbers and dampens the IL-33 responses of ILC2. **A** Flow cytometry gating strategy of ILC2 defined as live, singlet cells lacking lineage markers (LIN: CD11b, CD11c, CD3, CD19, NK1.1, FCeR1, Ter-1) and expressing KLRG-1 and CD127, as well as CD25, ICOS and IL-33R (data not shown for confirmatory markers). **B**, **C** Counts of ILC2 in the **B** BAL and **C** lungs are shown. **D** Viability was assessed for ILC2 in bronchoalveolar lavage (BAL) fluid and lungs during flow cytometry analysis. The data are shown as mean ± SEM and are representative of 4 independent experiments. **E**–**H** Cytokine expression by lung ILC2 cultured with IL-2, IL-7 and IL-33 for 3 days were determined by ELISA. **E** IL-5, **F** IL-13, **G** CCL3, and **H** CCL22 are displayed as pg/cell based on ILC2 count determined at the beginning of culture. Results are representative of 3 separate experiments with 4–6 animals included per group. As before, statistical significance was determined using one-way ANOVA with Tukey’s post-test as in previous figures. * Indicates *p* < 0.05; **p < 0.01, ***p < 0.001, ****p < 0.0001

ILC2 are important for allergic airway inflammation associated with asthma [17] and are increased in peripheral blood of asthmatic patients [50]. ILC2 are predominantly responsible for IL-5 and IL-13 production following in vitro and in vivo stimulation of mouse tissues or cells with IL-33 [51]. Importantly these cells have been shown to interact with type 2 helper T cells and to directly support eosinophil responses through their production of IL-5 [12–14]. First, we identified ILC2 as LIN- CD127+KLRG-1+ cells in the BAL and lungs following OVA challenge (Fig. 9A–C). We have reported sex differences in the activation of ILC2 with and without OVA challenge dependent on IL-33. In these studies, we detected estrogen-dependent differences in counts of BAL and lung ILC2 in OVX-E2-OVA compared to F-INTACT-OVA animals (Fig. 9A, B). We assessed viability of the ILC2 during flow analysis and confirmed that estrogen was not associated with lower viability in the BAL or lung ILC2 (Fig. 9D). Next, lung ILC2 were sorted from each treatment group for ex vivo culture with IL-33. We now add to our previous report by showing ILC2 from F-INTACT-OVA mice produced more IL-5, IL-13 CCL22 and CCL3 in response to IL-33 when compared to OVX-E2-OVA-treated mice. While the amount of IL-5 and IL-13 were higher in females compared to males in previous studies, we confirm here that the excessive production of these cytokines in females was not due to estrogen stimulation, as we found reduced IL-5 and IL-13 production, and reductions in pro-inflammatory chemokines, CCL22 and CCL3, by lung ILC2 obtained from OVX-E2-OVA compared to Female-INTACT-OVA-treated animals (Fig. 9E–H). IL-5, IL-13, CCL22 and CCL3 were all comparable in OVX-Pl-OVA and F-INTACT-OVA-treated animals.

## Discussion

In the present study, we examined the effect of steady-state estrogen in the well-defined OVA-induced allergic inflammation model. Substantial allergic inflammation was generated with 5 consecutive days of intranasal (i.n.) OVA administration. We then assessed pulmonary mechanics, innate immune populations in whole lung tissue and BAL, and type 2 inflammatory cytokine (IL-5 and IL-13) and chemokines (CCL17 and CCL22) detected in BAL and circulation. We leveraged this model to show how pulmonary mechanics changes occur in conjunction with E2 levels that correlated with the mid-follicular phase of ovulation [17β-E2 @ 68.2 ± 2 pg/mL]. We documented a significant reduction in ILC2 and eosinophils with this estrogen treatment and showed that ILC2 were reduced and became non-responsive to IL-33 stimulation when we isolated those cells from estrogen-treated animals. However, these reductions did not correlate with reduced airway resistance, as airway resistance remained higher in OVX-E2-OVA mice compared to F-INTACT-OVA-treated controls. Together, this indicates that estrogen is enhancing airway pathologic changes after allergen challenge, despite reducing the prototypical type 2 innate immune cells (i.e., eosinophils and ILC2).

Steady-state 17β-estradiol increased airway resistance and overall inflammatory scores following OVA challenge and PV-loops were flattened in estrogen-treated animals which indicates stiffening of lung tissues. There are clear airway structural changes occurring with estrogen treatment that resemble results from other studies [52]. Our interpretation of the data are that alveolar, airway epithelial and airway smooth muscle cells are directly stimulated by exogenous estrogen through estrogen receptors [52–56], with those studies in mind it is likely that the estrogen receptor alpha (ER-α) controls the airway smooth muscle responses to allergen. Estrogen receptor α knockout animals display spontaneous airway reactivity in early experiments that explored sex differences in asthma [57]. More recent work has shown that estrogen receptor α and estrogen receptor β activation have distinct consequences in animal modeling of allergen responses [58, 59]. Using an estrogen receptor β agonist precisely activates ER-β, and this report showed reduces airway resistance (Rrs) following allergen challenge. In that same study an ER-α agonist did not significantly alter Rrs in female or ovarectomized mice following allergen challenge [54]. In our studies, steady-state estrogen significantly increased Rrs generated in ovalbumin-challenged mice treated with 12.5 mg/mL of MCh to measure airway hyperreactivity. Comparatively, our study only challenged animals with a moderately low dose of methacholine, whereas Ambhore et al. [54] found the greatest effects with 50 mg/mL of methacholine. An additional comparison was the reduction in total BAL cells in our study and the reductions seen in a study that used the estrogen receptor β agonist [59]. Those animals given the ER-β agonist had reduced eosinophils, lymphocytes, and macrophages in response to mixed-allergen challenge. Our study similarly showed significant reductions in eosinophils, neutrophils, CD3+ T cells, CD19+ B cells, and ILC2 along the airways. While our model is likely activating both ER*-*α and ER-β in several cell types, it does suggest that ER-β may be responsible for the reduction of the airway immune populations. There are very few studies that examine ER-β responses, but data generated in non-allergic animal models do note a regulatory phenotype, or FOXP3+ T regulatory cell induction occurring via ER-β activation [60–63]. Perhaps regulatory T cells are decreasing inflammatory responses through direct (co-inhibitory molecules such as PD-1) or indirect immune inhibition (TGF-β, IL-10 production) [64, 65]. Of course, examining T regulatory cells was beyond the scope of the current study, but it is likely they are generated since OVA-induced allergic inflammation relies heavily of OVA-specific T cells.

One additional study documented a role for ER-α in the release of IL-33 from EpCAM+ airway epithelial cells [58], which indeed is an important factor in type 2 allergic responses. IL-33 induces substantial IL-5 and IL-13 responses from ILC2 [39]. Therefore, one would think that steady-state estrogen would increase the IL-33 response in the airways, however, we saw no changes in the amount of IL-33 in BAL or circulation with estrogen treatment; we furthermore, did not see a significant effect of biological sex on IL-33 production as previously report [66]. Although these conflicting results are likely due to various factors related to experimental design and method of IL-33 detection.

We did confirm a strong inhibitory effect of estrogen on the IL-33 responsiveness of ILC2 and showed that this decline in IL-5, IL-13 and chemokines was not because of reduced cell viability. It will be important to establish whether estrogen is inducing a different phenotype, perhaps the ILC3-like or an ILC regulatory phenotype, in future studies [67, 68]. Future studies will also investigate estrogen receptor expression specifically in ILC2. It has yet to be determined whether the estrogen receptors are expressed at the protein level in ILC2, although it was shown that lung ILC2 were not regulated by ER-α [58], and sequencing data showed low read counts for Esr1 (ER*-*α) and Esr2 (ER-β) transcripts in BM ILC2 [69]. Contrary to these reports, Bartemes et al., showed that uterine ILC2 were responsive to IL-33 and depleted in ER-α and ER-β knockout animals [70]. This suggests that tissue localization is important for estrogen responses involving inflammatory stimuli. Certainly, the uterus is directly exposed to fluctuations in ovarian hormones, and this may influence the expression of various hormone receptors over time. This is a critical gap to fill in allergy and asthma studies as many studies don’t verify protein expression of hormone receptors, nor do they examine changes in receptor expression during various activating stimulation (i.e., following IL-33 activation) [71]. To date, it is not yet established whether human ILC2 express the estrogen receptors at the protein level, but a recent clinical report defined increased circulating ILC2 in pregnant women compared to non-pregnant women. These data were correlated within each subject with higher levels of progesterone and estrogen in circulation of early (first trimester) and late pregnant (third trimester) women [72]. Together this indicates that the ovarian hormones are strongly associated with expansion of ILC2 in females.

## Perspectives and significance

The current study showed an estrogen-associated reduction in traditional allergic inflammation, despite increased airway resistance and mucus production, that were the likely by-product of neutrophil-associated inflammation generated by repetitive airway challenges. This reduced ILC2 and eosinophil responses were what we hypothesized based on the clinical studies that showed improvements in asthma severity with oral contraceptives. However, what was unexpected was the increase in airway hyperreactivity and inflammation noted in multiple areas in the lung, beyond just the airway cells. Together this study indicates that lung airway smooth muscle cells, and potentially alveolar cells and macrophages, become hyper-reactive because of estrogen. This again highlights the complicated nature of hormonal responses and their role in amplifying allergic responses in the lung. Future studies will continue to tease apart these interactions, between the various immune cells and lung cells in the context of hormonal fluctuations, to work towards identifying precision medicine strategies for male and female asthmatics.

## Data Availability

All data are present in the main text of the manuscript, and saline control data are available at request; the values from the various protein assay for the saline-treated animals were not detectable and removed to concisely show the relevant results in the paper.

## Abbreviations

AHR: Airway hyperreactivity
BAL: Bronchoalveolar lavage
Ers: Respiratory system elastance
FEV_1_: Forced expiratory volume for the first second of exhalation
FVC: Forced vital capacity
ILC2: Group 2 innate lymphoid cells
IL-5: Interleukin 5
IL-13: Interleukin 13
F-INTACT: Female, sham-operated
MCh: Methacholine
OVA: Chicken egg ovalbumin
OVX: Ovariectomized
Rrs: Respiratory system resistance
Th2: Type 2 CD4+ helper T cells
Th17: IL-17+ CD4+ helper T cells
